# User Context Detection for Relay Attack Resistance in Passive Keyless Entry and Start System

**DOI:** 10.3390/s20164446

**Published:** 2020-08-09

**Authors:** Jing Li, Yabo Dong, Shengkai Fang, Haowen Zhang, Duanqing Xu

**Affiliations:** College of Computer Science and Technology, Zhejiang University, No. 38, Zhe-Da Road, Hangzhou 310027, China; zjujing@zju.edu.cn (J.L.); fsk119@zju.edu.cn (S.F.); zhwlm@zju.edu.cn (H.Z.); xdq@zju.edu.cn (D.X.)

**Keywords:** PKES, relay attacks, smartphone, context detection

## Abstract

In modern cars, the Passive Keyless Entry and Start system (PKES) has been extensively installed. The PKES enables drivers to unlock and start their cars without user interaction. However, it is vulnerable to relay attacks. In this paper, we propose a secure smartphone-type PKES system model based on user context detection. The proposed system uses the barometer and accelerometer embedded in smartphones to detect user context, including human activity and door closing event. These two types of events detection can be used by the PKES to determine the car owner’s position when the car receives an unlocking or a start command. We evaluated the performance of the proposed method using a dataset collected from user activity and 1526 door closing events. The results reveal that the proposed method can accurately and effectively detect user activities and door closing events. Therefore, smartphone-type PKES can prevent relay attacks. Furthermore, we tested the detection of door closing event under multiple environmental settings to demonstrate the robustness of the proposed method.

## 1. Introduction

The Passive Keyless Entry and Start system (PKES) has been widely installed in modern vehicles. The PKES offers ease of use to drivers by automatically activating the unlock procedure as they approach their vehicles without pulling out keys from their pockets. The traditional PKES requires a key fob to verify the user’s proximity to the car. New models of Tesla, Volvo, and Lincoln enable car owners to use their smartphones to enter and start their cars [1,2], and the use of “phone as a key technology” is increasing. This technology uses Bluetooth Low Energy (BLE) positioning to determine the owner’s proximity to the car. Despite the convenience, both conventional and smartphone-type PKES are prone to relay attacks [3,4].

Relay attacks on PKES can be achieved using relay devices. Police in the UK published a video showing how some accomplices used two relay devices to steal a car in 1 min [5]. In a classic relay attack, a pair of attackers relay messages between the vehicle and the key without deciphering or modifying them; consequently, the PKES mistakenly considers that the valid key is near, which could be hundreds of meters away from the car [3]. Attackers can enter and even steal the vehicle without the car owner’s awareness as relay attacks are difficult to detect; therefore, it is essential to take precautions and preventive measures.

Researchers have studied various methods to resist relay attacks. Researchers in [6,7] proposed the measurement of the round-trip time between the car and key as a countermeasure against relay attacks. Choudary et al. [8] presented a solution to introduce intentional noise in the communication between two authentic devices. However, their method is vulnerable to the problem of long cable where attackers connect the two authentic entities using a long cable instead of creating two separate communication channels. Choi et al. [9] proposed a sound-based proximity detection method by computing the similarity of the sounds around the car and the key. Therefore, the sound-matching method has limitations that require cars to be parked in a noisy environment where the audio recording devices can directly access the surrounding sound. These relay attack prevention methods focus on improving the proximity detection. Ranganathan et al. [10] concluded that various attacks could exploit the proximity verification method.

We propose a secure smartphone-type PKES model based on user context detection (UCD). The PKES model establishes a BLE connection between the car and the smartphone and prevents relay attacks by limiting the user context when the PKES unlocks or starts. Our approach is based on the following facts. The key is usually idle during a relay attack on the PKES. Furthermore, the driver typically walks toward the car before unlocking it, and the driver is often inside the vehicle before pressing the start button. Therefore, our method needs to detect states such as walking, the car owner inside the car, and other human activities. These contexts can be detected by the accelerometer and barometer embedded in smartphones.

Our contributions are summarized as follows.

- We propose a secure smartphone PKES model. A growing number of manufacturers are introducing keyless systems that use smartphones as keys. Therefore, it is essential to protect smartphone PKES from relay attacks.
- We propose a user-context-based method to resist relay attacks. The proposed method resists relay attack by limiting the car owner position semantics when the car is set to unlock and start. Therefore, this method is not easily influenced by the environment, for only user behavior is concerned.
- We design a UCD algorithm based on the accelerometer and barometer embedded in smartphones. Extensive evaluations on our datasets reveal that the algorithm can effectively and accurately identify user state.

The rest of the paper is organized as follows. Section 2 discusses the related work. The background of using a smartphone as a PKES key is presented in Section 3. Section 4 details how our method can resist relay attacks on PKES. Data collection and experiments are discussed in Section 5. Section 6 discusses the limitations of our work and details our future work. Section 7 provides the conclusion.

## 2. Related Work

### 2.1. Passive Keyless Entry and Start System

Waraksa et al. [11] described PKES, which can automatically unlock the vehicle when the driver carrying the corresponding key approaches the car and locks the vehicle while the key is out of the range to the vehicle. The system does not require any action from the driver and automatically verifies the corresponding key when the key is nearby using a short-range communication channel.

Typically, the traditional PKES adopts a key fob containing a low-frequency (LF) radio frequency identification (RFID) tag and ultrahigh-frequency (UHF) transceiver, and the vehicle is equipped with a LF transceiver and UHF RFID tag. The LF channel is a short-range communication channel used by the vehicle to detect if the key fob is close to the car. The vehicle sends messages on the LF channel, and the corresponding key responds based on the UHF channel.

In recent times, the new PKES replaced the key fob with a smartphone. Karani et al. [12] and Lin et al. [13] designed and implemented the PKES based on BLE. In the new PKES, the vehicle determines if the key is in proximity and unlocks itself by monitoring the BLE received signal strength indicator (RSSI) measurements from the key.

### 2.2. Relay Attacks

Researchers analyzed and verified relay attacks against PKES systems [3,4]. Francillon et al. [3] successfully unlocked a car by amplifying and relaying the key signal to the vehicle; thus, the vehicle could sense the key fob signal even if it is far away. Distance bounding techniques are commonly proposed to prevent relay attacks [14,15,16]. These techniques require the prover to rapidly respond to the challenges from the verifier as the verifier calculates an approximate distance from the verifier to the prover by the elapsed time used to complete the challenge–response process. The communication stack needs to be smaller; otherwise, a small transfer error can lead to significant deviations in the distance estimation. Furthermore, context-based detection methods are used to prevent relay attacks, which validate the proximity by calculating the similarity between the environment of the car and that of the key [17,18,19]. Therefore, researchers utilized WiFi, Bluetooth, and ambient sound for proximity detection [9,20,21]. However, the key and the vehicle are required to collect data from the ambient environment and compute the similarity of the features. Their methods focus on two entities (the key and the vehicle), so the algorithm is executed on the car and the key and requires the installation of relevant sensors in the car. This paper proposes a UCD algorithm which only focuses on the user behavior to verify unlock and start commands and only runs on the valid user’s smartphone, making it less susceptible to the environment.

### 2.3. Detecting Activities Using Smartphone Sensors

Research has been done in activity recognition using sensors for several years, and the methods differ in choosing and deploying sensors, the type of activities detected, and computational activity models. An extensive survey is presented in [22]. As the smartphone is equipped with various sensors, the implementation of activity recognition on the smartphone is gaining attention [23]. The accelerometer is the dominant sensor used for activity detection. Researchers in [24,25,26] used the accelerometer of the smartphone to determine transportation models. Ryder et al. [27] revealed that the Global Positioning System (GPS) could support the accelerometer in improving the accuracy of the transportation model detection. Barometers are introduced into smartphones for aiding GPS [28], and then smartphone barometers are used for human activity recognition and context awareness [29,30]. The detection of activities using smartphone sensors is a classic multivariate time series classification problem that extracts discriminative features from sensor data and chooses an appropriate classifier to recognize activities. With the rapid development of deep learning, it has been introduced in human activity recognition, which uses the deep neural network as an automatic feature extractor and classifier [31,32].

## 3. Background

As described in the introduction, we need to detect user context to resist relay attacks. The user contexts include walking, the state that the car owner is inside the vehicle, and other activities. The use of smartphones to recognize human activity has been widely studied by various researchers, as presented in related works. Therefore, we will describe how to use a smartphone to detect the state of the driver inside the car.

Based on the empirical evidence that the driver closes the door after entering the vehicle, and the door is closed when the car starts, utilization of the user’s smartphone to detect door closing events inside the vehicle indicates that the user is in the car. The use of a smartphone to detect door closing is based on the following observations.

**Figure 1:**
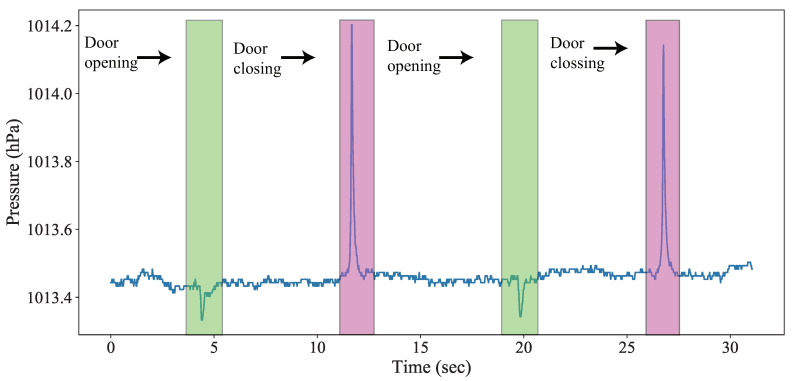
Atmospheric pressure variation due to the vehicle door opening/closing events.

**Observation-1:***The smartphone barometer can detect car door closing events.*Figure 1 shows that the smartphone barometer records the atmospheric pressure variation caused by the events of the car door opening and closing. We observed that the atmospheric pressure inside the car decreases and then rapidly returns to normal when we open the door; consequently, we can identify distinct atmospheric pressure troughs when the door opens. The atmospheric pressure decreases when we open the door because the air inside the car becomes thinner and then rapidly restores to normal because it is not completely sealed. Conversely, the air is pushed into the car when we close the door, increasing the air pressure in the car and then rapidly restoring to normal. Therefore, we can identify the atmospheric pressure spike when we close the door. The smartphone barometer can sense the car door opening and closing events. Although the atmospheric pressure is affected by drift due to weather, Sankaran et al. [29] proved that windy weather does not produce significant drift in the barometer. This is due to windy weather or a sudden gust of wind that lasts a long time which is much longer than the sliding window (2 s) in our method, and weather drift is usually in one direction. A sudden gust of wind cannot create an atmospheric pressure spike or trough in 2 s.

**Figure 2:**
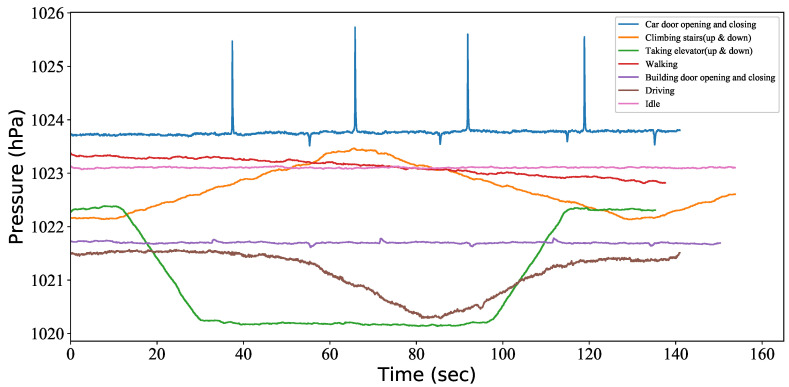
Atmospheric pressure variation due to user activities.

**Observation-2:***The characteristics of the pressure variation caused by car door closing are different from that of the building door shutdown.*Figure 2 presents the pressure variation indoors due to the closing of the building door. Even though the pressure variation caused by building door events also has spikes and troughs, the rate of pressure change due to car door closing events is much faster than that of the building door. The barometer can distinctly recognize the car door events and the building door events using the appropriate features.

**Observation-3:***The method only using the barometer is unable to differentiate the activity used to resist relay attacks.*Figure 2 presents the barometric change detected by the barometer on the smartphone carried by the user during their daily activities. We observed that the barometer could differentiate the activity with significant changes in height. However, the pressure variation pattern for walking on a flat floor is similar to when the smartphone is idle. Therefore, only using the barometer is not sufficient to recognize different human activities. When the PKES has detected that the smartphone is within the BLE communication distance, the relay attack prevention method requires input from an accelerometer to ensure the user is walking toward the car.

## 4. Methodology

In this section, we will discuss in detail the smartphone-type PKES model and describe how the UCD method can protect the PKES against relay attacks.

### 4.1. System Model

The system model adopts the idea of using a phone as a key technology. In our system model, the car verifies its corresponding smartphone’s proximity using BLE positioning. The communication between the car and its corresponding mobile phone is based on the protocol in [13]. Apart from the proximity detection, the novelty of our system is that it verifies the reasonability of user context when the car considers that the smartphone is nearby.

Unlike the existing PKES, which has only one verification for entering and starting the vehicle, our system utilizes the UCD algorithm to determine if it is rational to execute entry and start commands.

#### 4.1.1. Entry Verification

**Figure 3:**
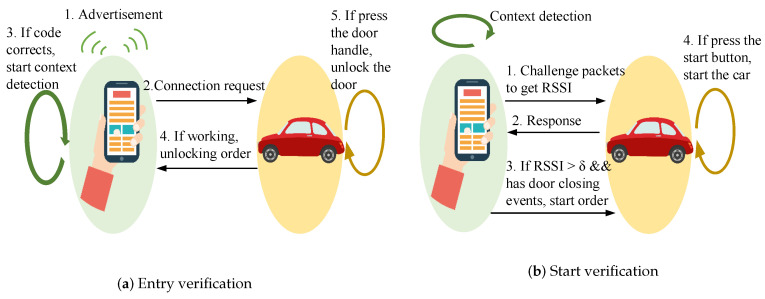
Smartphone-type Passive Keyless Entry and Start system (PKES).

As analyzed earlier, walking is the only rational state when the user wants to unlock. Therefore, the entry verification uses UCD to detect the user context and determine whether the user is walking when he/she is in the BLE communication range. To explain the entry verification process, we detail each procedure used by the car to verify its corresponding phone based on proximity verification and UCD, as presented in Figure 3a.

1. The smartphone periodically broadcasts BLE advertisements with authentication information.
2. If the car is within the BLE communication range, it sends a connection request to the smartphone.
3. If the connection request packet carries the correct verification code, the smartphone starts context detection.
4. If the smartphone is not in the walking state, the PKES stops sending an unlocking packet. Otherwise, the smartphone sends an unlocking packet to the car.
5. After the car receives the unlocking order, the car automatically unlocks the door when the user presses the door handle.

#### 4.1.2. Start Verification

Even though entry verification can prevent most relay attacks, the attacker can bypass the entry verification by stalking the potential victims. In that case, the attacker will successfully drive away if the PKES has no start verification. Start verification is used for determining whether the person who started the vehicle is a legitimate user. As analyzed in Section 3, the detection of a door closing event can prove that the car owner’s phone is inside the car. Therefore, the start verification requires the use of UCD to determine if there is a car door closing event. Figure 3b presents the detailed start verification process.

1. The smartphone performs context detection and periodically sends a challenge packet to the car to get the current relevant distance.
2. The car sends a response packet to the smartphone after receiving the challenge packet.
3. If the RSSI of the response packets from the car is larger than the predefined threshold, indicating that the smartphone is inside the car, and the UCD algorithm has detected a door closing event, then the smartphone sends the start command to the car.
4. If the user presses the start button, the car automatically starts the ignition.

### 4.2. UCD Algorithm

**Figure 4:**
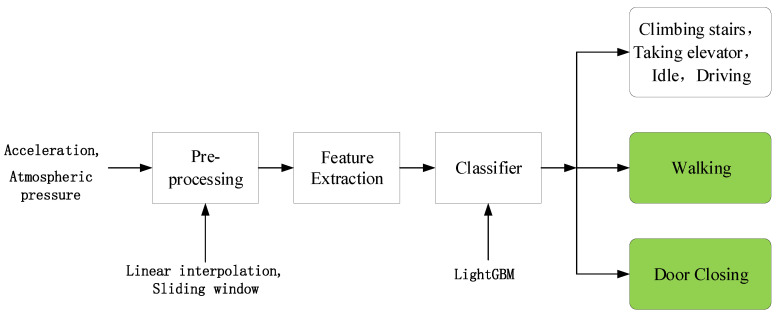
Overview of the UCD algorithm.

The UCD algorithm enables smartphone-type PKES to achieve entry verification and start verification. Unlike other context detection algorithms, the UCD requires the detection of the driver’s daily activity, including walking, door closing, climbing stairs, taking an elevator, and idle. Note that the state “climbing stairs” includes when the user is both on the stairs and on the landing area; both states indicate that the user is on the stairs and cannot unlock the vehicle. Figure 4 presents a high-level overview of the UCD.

#### 4.2.1. Preprocessing

The data delay of the barometer and accelerometer is set to SENSOR_DELAY_GAME to increase accuracy and energy efficiency [33]. In phones such as Mi 5, values are returned at a higher rate, usually every 0.02 or 0.03 s. Consequently, the original sample is converted to 50 Hz using linear interpolation.

Considering that the segmentation window size influences the user context’s recognition performance, we choose an appropriate window size to accommodate different activities. We divided the events into two groups: instant and long-duration activities. Instant activities rapidly take place, such as a door closing event which continues only for 1 or 2 s. Long-duration events are repetitive and proceed for a long time, much longer than the instant activities, such as walking, taking an elevator, and climbing stairs. These activities often extend beyond 2 s. Based on the experience of selecting window size for long-duration activities in previous studies [29,34,35], we set the sliding window size to 2 s. A window size of 2 s is efficient and adequate to accommodate the door closing event. Moreover, we set 1 s for the overlap of the sliding window.

#### 4.2.2. Feature Extraction

We studied various features and extracted the most promising features from the sliding window of 2 s. We use $X=[x_{1},x_{2},\cdots ,x_{100}]$ to denote the sensor data of one sliding window. The accelerometer and barometer data are denoted as $X_{a}$ and $X_{p}$, respectively. We have extracted 12 features, as presented in Table 1.

#### 4.2.3. Training

We choose the LightGBM as our classifier, which is a gradient boosting framework that uses tree-based learning algorithms [36]. We selected the LightGBM as it can deal with multiclass classification problems on unbalanced datasets. In LightGBM, we train six one-versus-all classifiers over six activity classes and generated the final score of each class by normalizing the score of all classifiers. Therefore, the multiclass classification problem on imbalanced datasets is transformed into a binary classification problem on unbalancing datasets. For each binary classification, the weighted objective function is introduced into the classifier to calculate weight by the ratio of each class in the training dataset.

**Figure 5:**
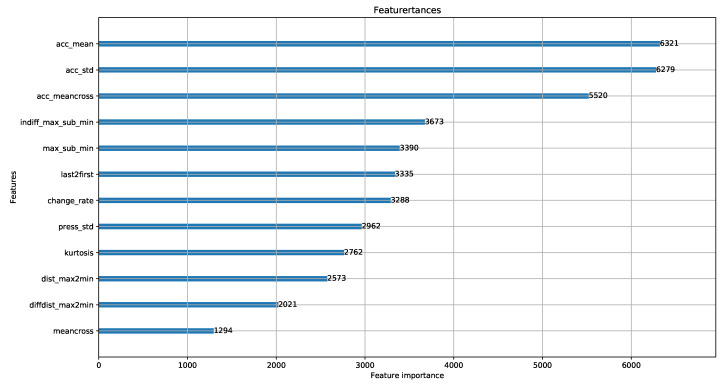
Feature importance.

In the UCD, the LightGBM is trained by the features described in Section 4.2.2. After training, we evaluated the feature importance of the UCD by LightGBM. As presented in Figure 5, the acceleration feature is of utmost importance in our algorithm as multiple user activity, which needs to be identified to prevent relay attacks, takes place on the horizontal ground.

Wu et al. [34] used a barometer to differentiate building door events from other user activities. Their algorithm only uses three barometric features (mean cross, change rate, and standard deviation) to train the classifier. To test if the algorithm only trained by the above barometric features can be applied to resist relay attack, we also train the LightGBM with these barometric features and analyze it in our dataset. We denote this algorithm as the monitoring door events (MDE) algorithm in subsequent analysis.

## 5. Experiments and Analysis

In this section, we first explain the implementation of the model and the devices we use in the experiment. Second, we describe our method for data collection. Subsequently, we evaluate the performance of the position verification method.

### 5.1. Experimental Setup

#### 5.1.1. System Implementation and Devices

We develop the system model application and implement it on two Mi 5, one Mi 4, and one Mi 2s. Each smartphone is equipped with a barometer and an accelerometer. The details of the phones are presented in Table 2. Table 2 shows that the mobile phones used in the experiment are obsolete with lower sensor configuration than newer models. We assume that modern smartphones can resist relay attacks based on the capability of these outdated ones, and also there are often hardware improvements in the latest smartphones. Moreover, two different types of cars are employed in the experiment, and the details of the cars are presented in Table 3.

**Figure 6:**
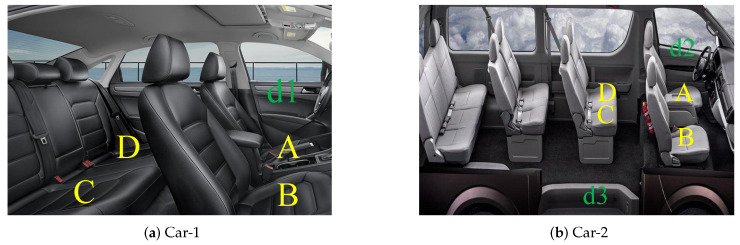
Car interior plan and locations of closing doors.

Note that the significant differences between the two cars are the interior volume, the type of doors, and the fact that car-2 has one sliding door and two regular doors and car-1 has four regular doors. d1 and d2 are regular doors and d3 is a sliding door, as presented in Figure 6.

#### 5.1.2. Data Collection

We evaluated our proposed method based on the measurements of the accelerometer and barometer collected from four Mi smartphones, which are described in the above section. Five participants were recruited to carry the smartphone to collect the driver’s daily routine data, including walking, taking an elevator, climbing stairs, driving, idle, and closing the car door. They need to record the type and time of the activity. The idle state implies that the user is not moving or not carrying the smartphone.

When the participant is outside the car, the orientation and position of the smartphone is not limited. The phone can be put in a bag or pocket or can be held in hand. When the participant is inside the car, the orientation of the phone remains unrestricted; however, smartphones need to be placed on different seats, as presented in Figure 6.

Considering that the smartphone’s position in the car and other factors may influence the detection of the door closing events, we conduct the door closing experiments in different scenarios as follows.

**Task-1:** The purpose of Task-1 was to observe whether the UCD algorithm can detect regular door closing events in any seat. In each car, we asked four participants to sit in A, B, C, and D. The participant sitting in A opened and closed d1 or d2 repeatedly, and one of the other participants recorded the time of closing the door. The rest of the tasks also need to record the closing time.

**Task-2:** The purpose of Task-2 was to explore whether the sliding door closing events can be detected in any seat. Note that the sliding door only exists in car-2, so we completed Task-2 only in car-2. The position of the participants is the same as that in Task-1; the difference is that the participant sitting in C was required to open and close d3.

**Task-3:** The work in [34] found that monitoring building door events using the barometer in smartphones is required in an insulated environment, and Mahler et al. [37] demonstrated that the method in [34] will be ineffective if the home has an open window. Therefore, we studied the applicability of our UCD when the car window is opening and how the window’s opening size affects the performance. The experimental set-up is the same as Task-1 in terms of the position of the participants and the door we were closing. We conducted the door closing experiments with the window opened below 10 cm in car-1.

We collected 1223 door closing events, and the number of other activity samples is presented in Table 4. We divided the dataset into training and test sets. For the door closing event, we randomly selected two-thirds of the door closing samples with windows no larger than 2 cm from car-1 as the training set and other door closing samples as the test set. For the samples of other activities, we randomly selected two-thirds of each activity as the training set and one-third as the test set. Moreover, we used the fivefold stratified cross-validation technique on the training dataset to determine the optimal hyperparameters.

### 5.2. Evaluation

We used the following performance metrics to evaluate our method’s accuracy, precision, recall, F-measure, and confusion matrix, as presented in Table 5. In Table 5, $n_{ij}$ denotes the number of test samples of the activity *i* classified as the activity *j*. We split each activity’s timeline into 2 s intervals and checked whether the context of interval determined by the position verification method matches the ground truth. One interval represents an activity sample.

#### 5.2.1. Evaluation for Recognizing User Context

To determine the effectiveness of our method in resisting relay attacks, we analyze the UCD algorithm’s capability to identify walking, door closing, climbing stairs, taking an elevator, idle, and driving states. This section focuses on regular door closing events from a sedan (car-1) because PKES systems are mostly used in sedans. The influence of car type and door type will be discussed in the next section.

Table 6 presents the detection results of the proposed algorithm for different activities. The algorithm performed best in detecting a door closing, with a precision and recall of 99.8% and 97.0%, respectively. This performance is due to the use of the barometer in the algorithm, which senses the slight pressure changes inside the car caused by the door closing. It also performed well in detecting the states of taking an elevator, idle, and driving with accuracy and recall above 90%. In terms of Walking and Climbing stairs identification, UCD performs poorly, and the F-measure is below 90%. The recall of Climbing stairs is only 50.6%.

Table 7 presents the confusion matrix for the UCD algorithm, which provides insight into the potential cause of the low recognition rate of walking and climbing stairs. It is easy for our algorithm to mistake climbing stairs for walking (33.9%) and driving (15.5%). Moreover, 3% and 4% of walking samples are misclassified as climbing stairs and driving, respectively. Four factors contribute to these phenomena: First, the walking speed can vary significantly, depending on multiple factors, such as height, age, terrain, and surface. When the participant walks quickly on a ramp, the change in air pressure and acceleration is similar to climbing stairs and taking an elevator. Second, our method only uses the features in the time domain to reduce the computational complexity of the algorithm. Compared with the recognition methods of other activities that use both features from the time and frequency domains, our approach achieved a lower detection rate in some activities. However, it performed satisfactorily on critical context detection (door closing events). Third, during data collection, the walking terrain contains flat and sloped roads, and the data of climbing stairs include walking on the staircase landing area. Therefore, some samples of walking and climbing stairs are essentially the same but belong to different contexts. Fourth, due to the short sliding window used during the detection, the data of walking on the staircase landing area are divided into several 2 s climbing stair samples, which are wrongly classified as walking.

#### 5.2.2. Influence of Feature on the Context Detection

**Figure 7:**
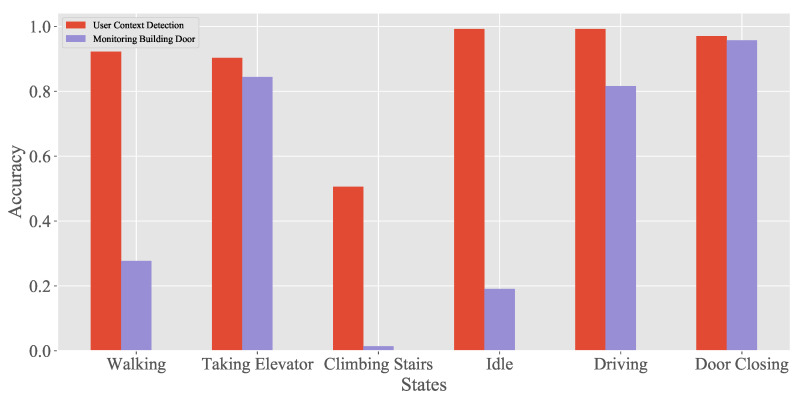
The detection comparison between the UCD and the monitoring building door (MBD).

Similar to our work, Wu et al. [34] used the barometer sensors of smartphones to monitor building door events. In this section, we explore the features in [34] and consider their potential to prevent relay attacks. For comparison, we used the LightGBM as the classifier for the monitoring building door (MBD) algorithm and our datasets as the training and testing sets, as mentioned in Section 4.2.3. Figure 7 presents the comparison results of the two algorithms. Because barometers are sensitive to atmospheric pressure changes inside the car, the MBD performed well in detecting door closing events with 95.7% accuracy. The accuracy of UCD is slightly higher than that of MBD due to the use of acceleration and atmospheric pressure features. However, the MBD performed poorly in the detection of other activities. The accuracy of climbing stairs detection was below 10%.

Table 8 presents the confusion matrix of the MBD. We observed that 56.7% and 15.1% of walking samples are incorrectly classified as driving and idle events, respectively. Furthermore, 82.4% of climbing stairs samples are wrongly classified as driving. The MBD often misclassified other events, except for the door closing event. There are two reasons for the low detection rate of the MBD algorithm: First, a barometer detects context based on the phenomenon that atmospheric pressure changes with altitude [29]. However, to prevent relay attacks, our dataset covers multiple types of terrain encountered during daily driving and car pickup, including roads with potholes and flat roads in walking samples. The height of the phone slightly changes when the user walks on flat ground. Therefore, training the algorithm using only barometric features often leads to the misclassification of walking and idle events.

#### 5.2.3. Optimization for Recognizing Long-Time Activities

From the analysis in the previous section, we observed that the 2 s sliding window size is sufficient for short-time event detection, such as the door closing event detection. However, such a window size is insufficient for long-time activities, such as climbing stairs. The long-time event consists of irregular repetitive patterns, such as the user rising slowly at some steps. Moreover, the duration of long-time activities is also not fixed, so extending the sliding window size can only be useful for some samples with duration time approximately equal to the window size. Nevertheless, the window size cannot be infinitely increased. The larger the sliding window size, the less accurate the timing of the door closing event, which can impact the result of detection when the user is inside the car. Therefore, we use a simple rule-based algorithm to optimize the detection performance for long-time activity, as presented in Algorithm 1.
**Algorithm 1:** Simple rule-based algorithm.
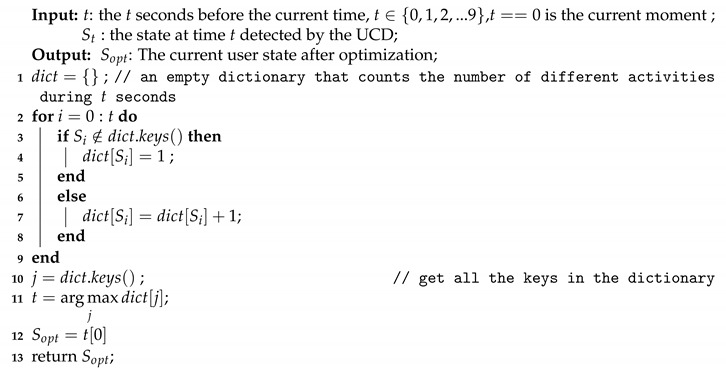


**Figure 8:**
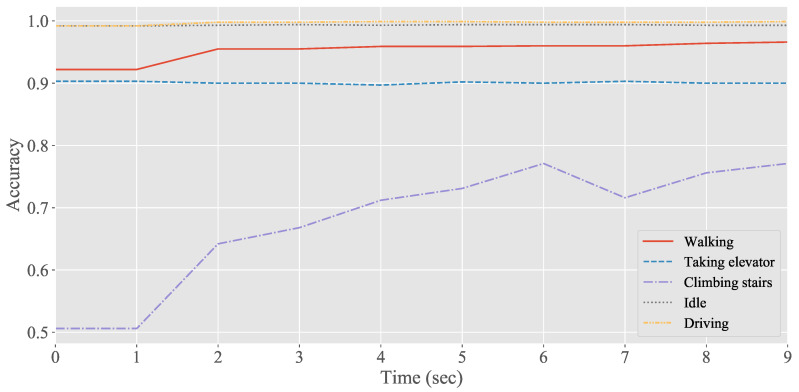
Detection performance for long-time activity after optimization using the simple rule-based algorithm.

Figure 8 presents the detection performance for long-time activity after using the simple rule-based algorithm. This optimization led to a significant enhancement of detection for climbing stairs, improving the accuracy from 50.6% to 77.1%. Combined with the previous activity, most of the samples of walking on the staircase landing area are correctly classified as climbing stairs. Moreover, using 6 s of the previous information and combining the past context semantics increase the accuracy of climbing stairs detection. However, the accuracy of climbing stairs detection combined with the context semantics of the past 7 s is poor compared with that of the previous 6 s. The rule-based algorithm introduced semantic information from the previous states; therefore, the transition instance is vulnerable to latency, which is one of the limitations of the proposed method. The optimization algorithm has little improvement in detecting the state of taking an elevator because it is a continuous process, and there is no transition instance to be optimized. Moreover, the data of the state of taking an elevator is collected from a six-story building with each lift ride taking less than 20 s. Therefore, the state of taking an elevator is less affected by other states because the movement speed differs significantly from those of other activities. The accuracies for the driving and idle states have slightly improved after using the simple rule-based algorithm.

### 5.3. The Robustness of the UCD Algorithm

Unlike walking and other activities, the changes in atmospheric pressure and vibration caused by the door closing events are based on the car’s configuration. To analyze the effect of the car’s configuration, we separately collected door closing events with different phone locations, door types, and open window sizes from two kinds of cars. In this section, we review the impact of these factors.

#### 5.3.1. Influence of Car Type and Phone Position

As mentioned earlier, the training dataset of the UCD algorithm is only from a sedan (car-1). To verify the effectiveness of our algorithm on other types of cars, we collected data of the door closing events from a minibus (car-2). The UCD algorithm ensures that the valid user is in the driver’s seat to prevent relay attacks on the passive keyless start; therefore, this experiment targets the door closing event of d2 (next to the driver’s seat).

**Figure 9:**
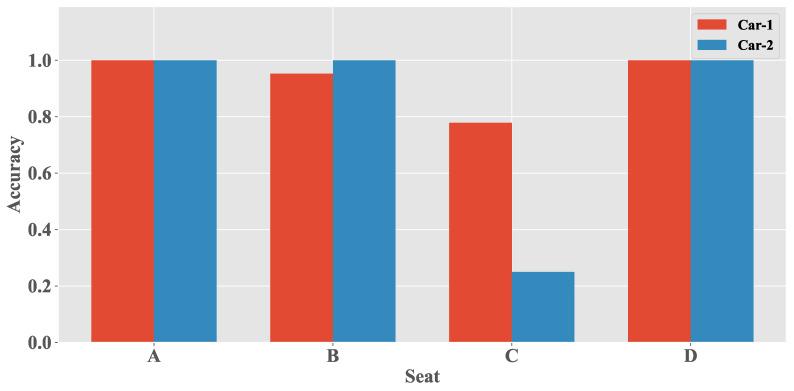
Door detection performance in different kinds of car.

Figure 9 presents a comparison for detecting door closing events on different vehicles. As can be seen from the figure, when the phone is in the driver’s seat, the accuracy of detecting the door closing event in sedan and minibus is high. This is because the door is close to the driver’s seat, which causes significant atmospheric pressure changes around the driver’s seat as the door closes. Moreover, the algorithm performed well in seats B and D with accuracy above 95%. However, it performed poorly when the phone was in seat C, achieving 77.8% and 25.0% accuracy in the sedan and minibus, respectively. The atmospheric pressure around the position farther from d2 changes slightly because the car is not completely sealed. For seat C, note that the accuracy of the door closing event detection is better in car-1 (77.8%) than in car-2 (25.0%). The atmospheric pressure around seat C of car-1 changed significantly compared with that of car-2 because the interior space of car-1 is smaller than that of car-2.

#### 5.3.2. Influence of Door Type

In the prevention of relay attacks on PKES, the detection of the closing of other doors can also support the identification of when the user is in the car. Note that the vehicle is symmetrical. Therefore, we can infer the detection performance for the closing event of other regular doors from the detection results of d1 in Section 5.3.1. In this section, we explore the effect of door type on closing detection.

**Figure 10:**
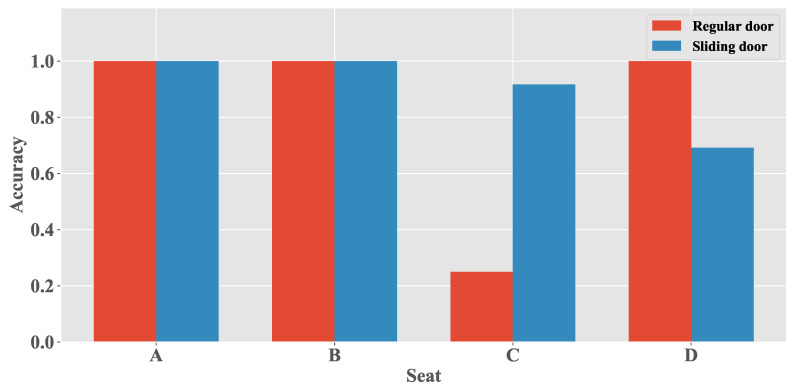
Door detection performance with different type of doors.

For fairness of comparison, we have implemented this experiment in car-2 because it has both regular doors and a sliding door. Figure 10 presents the detection results of the door closing events for different types of doors. The detection rate of the door closing event caused by closing the regular door or the sliding door in these two positions is high because d2 and d3 are close to seats A and B. The detection rate of the sliding door’s closing events is higher than that of the regular door when the phone is placed in seat C, whereas in seat D, the two are opposite. This is because the regular door is near seat A, and the sliding door is near seat C. Therefore, the detection of the door closing events is independent of the type of door being closed; however, it is related to the distance between the smartphone and the closing door.

#### 5.3.3. Influence of Opening Window

Mahler et al. [37] revealed that the use of the smartphone barometer to monitor building door events [34] becomes invalid when the window is open. Our strategy uses a smartphone barometer; therefore, we analyzed in detail the effect of having an open window on the proposed algorithm.

**Figure 11:**
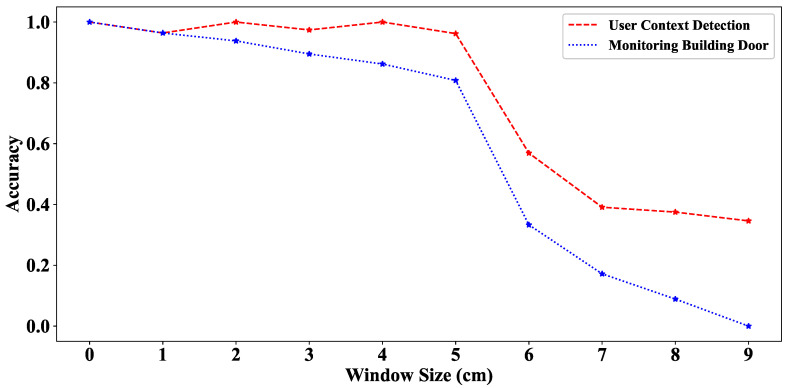
Door detection performance under different opening window size.

Figure 11 presents the accuracy of the door closing event detection of the UCD and MBD algorithms using the LightGBM classifier at different window opening sizes. We observed that the UCD outperformed MBD in detecting door closing events in all cases. When the window was opened less than 5 cm, both algorithms performed well in detecting door closing events at the driving position, with an accuracy of over 90%. Conversely, when the window was opened more than 5 cm, the recognition accuracy of the two algorithms was reduced. However, the UCD is less affected by having an open window compared with the MBD because our approach uses the features of accelerometer and barometer. When the windows are wide open, the car’s vibration caused by closing the door plays a significant role in the detection of the door closing event.

## 6. Discussion and Future Work

Our results show that the UCD algorithm can effectively identify if the user is inside the car, walking, or performing other daily activities. These characteristics are used to prevent relay attacks on PKES systems. This relay attack prevention technique assumes that the car owner is walking toward the car when the car receives an unlocking command and the car owner is inside the vehicle when the car is instructed to start. However, this approach has inherent limitations.

An attacker can successfully unlock the car by following the car and carrying out the relay attack on the PKE system as soon as the car owner leaves the car. However, the attacker cannot start the car as the owner’s smartphone has not detected a door closing event. We will combine the pedestrian dead reckoning [38] technique to detect the user walking direction in our future work. Consequently, users can unlock the door only when they are walking toward the car.

Furthermore, the experimental results show that the proposed approach can efficiently capture door closing events when the window is opened less than 5 cm. The door closing event detection can fail if the user leaves the window wide open for a while and then restarts the vehicle. To tackle this issue, we plan to utilize the GPS or the user moving trajectory based on the positioning techniques [39] to detect whether the user is inside the car or not. Moreover, with the installation of an automatic window closing system in most modern vehicles, the state wherein the driver leaves the car with the window open will seldom occur.

## 7. Conclusions

This paper reviewed the threat of relay attacks to the PKES systems and proposed a secure smartphone-type PKES system model. This system establishes a connection between the car and its corresponding smartphone using BLE. It utilizes a UCD algorithm to verify the environment semantics before the user enters and starts the vehicle. The UCD leverages the accelerometer and barometer embedded in smartphones to detect daily user activities and car door closing events. We verified the algorithm on the dataset composed of the drivers’ daily activities and 1526 door closing events under different scenarios. The results show that the UCD can accurately and efficiently identify walking and door closing events on the sedan, achieving accuracies of over 92% and 97%, respectively. For the door closing event detection, the UCD model applies to different types of vehicles without the need to retrain the model. Notably, the UCD can detect a door closing event even if the window is open. Furthermore, we discussed several approaches applicable to our method to deal with the situation where the assailant follows the car owner.

## Figures and Tables

**Table 1:** Features used in the user context detection (UCD) algorithm.

Features	Representation	Description
press_std	$\delta =\sqrt{\frac{1}{99}\sum_{i=1}^{100}{\left(x_{Pi}-\mu \right)}^{2}},\mu =\frac{1}{100}\sum_{i=1}^{100}x_{Pi}$	Standard deviation of atmospheric pressure fluctuations of the window
kurtosis	$g_{2}=\frac{\frac{1}{n}\sum_{i=1}^{n}{\left(x_{Pi}-\mu \right)}^{4}}{{\left(\frac{1}{n}\sum_{i=1}^{n}{\left(x_{Pi}-\mu \right)}^{2}\right)}^{2}}-3$	Kurtosis of atmospheric pressure fluctuations of the window
last2first	$X_{P100}-X_{P1}$	The difference between the last and the first atmospheric pressure in the window
max_sub_min	$Max\left(X_{P}\right)-Min\left(X_{P}\right)$	The difference between the maximum and minimum atmospheric pressure in the window
indiff_max_sub_min	$Max\left(diff\_X_{P}\right)-Min\left(diff\_X_{P}\right),$ $diff\_X_{P}=X_{Pi}-X_{Pi-1},i=2,3,\ldots 100$	The difference between the maximum and the minimum of a difference in atmospheric pressure
diffdist_max2min	$|index\left(Max\left(diff\_X_{P}\right)\right)-index\left(Min\left(diff\_X_{P}\right)\right)|$	The index distance between the maximum and the minimum of the difference in atmospheric pressure
change_rate	$Max(|x_{Pi+25}-x_{Pi}|),i=1,2,\ldots 75$	The rate at which the atmospheric pressure changes during the window
meancross	$Count\left(i\right),\exists i,\left(x_{Pi}\leq \mu \leq x_{P(i+1)}\right)\vee \left(x_{P(i+1)}\leq \mu \leq x_{Pi}\right),$ i=1,2,…99	The number of times observed atmospheric pressure crosses the mean pressure of the window
dist_max2min	$|index\left(Max\left(X_{P}\right)\right)-index\left(Min\left(X_{P}\right)\right)|$	The index distance between the maximum and minimum of the atmospheric pressure
acc_mean	$\mu_{A}=\frac{1}{100}\sum_{i=1}^{100}x_{Ai}$	The mean of the acceleration during the window
acc_std	$\delta_{A}=\sqrt{\frac{1}{99}\sum_{i=1}^{100}{\left(x_{Ai}-\mu_{A}\right)}^{2}}$	The standard deviation of the acceleration during the window
acc_meancross	$Count\left(j\right),\exists j,\left(x_{Aj}\leq \mu \leq x_{A(j+1)}\right)\vee \left(x_{A(j+1)}\leq \mu \leq x_{Aj}\right),$ j=1,2,…99	The number of times observed acceleration crosses the mean acceleration of the window

**Table 2:** The detail of smartphones.

Phones	Barometer Chip	Power/μA	Resolution / hPa
Mi 2s(2013)	Bosch BMP 180	3	0.01
Mi 4(2014)	Bosch BMP 180	3	0.01
Mi 5(2016)	Bosch BMP 280	2.74	0.01

**Table 3:** The detail of experimental cars.

Cars	Honda(car-1 )	Brilliance-Auto Jinbei Hiace (car-2)
Regular door	4	2
Sliding door	0	1
Number of seats	4	13
Length/mm	4212	5400
Width/mm	1742	1690
Height/mm	1531	2225

**Table 4:** Summary of samples for different activities.

	Walking	Taking Elevator	Climbing Stairs	Idle	Driving	Door Closing	Total Samples
Number	2362	2010	749	5060	10,965	1526	22,672

**Table 5:** Performance criteria.

	Definition	Formula
True Positive (TP)	The number of instances of the activity *i* which have been correctly classified	$Tp_{i}=n_{ii}$
False Positive (FP)	The number of instances of other activities which have been wrongly classified as activity *i*	$FP_{i}=\sum_{j,j\neq i}n_{ji}$
True Negative (TN)	The number of instances of other activities which has been correctly classified	$TN_{i}=\sum_{j,j\neq i}n_{jj}$
False Negative (FN)	The number of instances of activity *i* which has been wrongly classified as other activities	$FN_{i}=\sum_{k,i\neq k}n_{ik}$
Accuracy	The proportion of all instances which have been correctly classified	$Acc=\frac{\sum_{i}n_{ii}}{TP_{i}+FP_{i}+TN_{i}+FN_{i}}$
Precision	The fraction of instances predicted to the activity *i* which is correct	$Prec_{i}=\frac{Tp_{i}}{TP_{i}+FP_{i}}$
Recall	The fraction of instances of the activity i that are correctly predicted	$Reca_{i}=\frac{TP_{i}}{TP_{i}+FN_{i}}$
F-Measure	The harmonic mean of precision and recall	$2\frac{Prec_{i}*Reca_{i}}{Prec_{i}+Reca_{i}}$
Confusion Matrix	A confusion matrix *C* is such that $C_{i,j}$ is equal to the ratio of observations known to be in group *i* and predicted to be in group *j*.	$C_{i,j}=\frac{n_{ij}}{\sum_{j}n_{ij}}$

**Table 6:** The detection performance of the UCD.

Activity	Precision	Recall	F-Measure
Walking	86.2%	92.2%	89.1%
Taking Elevator	96.2%	90.3%	93.2%
Climbing Stairs	81.5%	50.6%	62.4%
Static	99.8%	99.2%	99.5%
Driving	90.3%	99.2%	94.5%
Door Closing	99.8%	97.0%	97.8%

**Table 7:** Confusion matrix for the UCD algorithm.

	Predicted	Walking	Taking Elevator	Climbing Stairs	Idle	Driving	Door Closing
Actual							
Walking		92.2%	0%	3.0%	0%	4.7%	0%
Taking elevator		0.5%	90.3%	0%	0%	8.9%	0.3%
Climbing stairs		33.9%	0%	50.6%	0%	15.5%	0%
Idle		0%	0%	0%	99.2%	0.8%	0%
Driving		0.5%	0.1%	0.2%	0%	99.2%	0%
Door closing		0%	0.6%	0%	0%	2.4%	97.0%

**Table 8:** Confusion matrix for the MBD algorithm.

	Predicted	Walking	Taking Elevator	Climbing Stairs	Idle	Driving	Door Closing
Actual							
Walking		27.7%	0.5%	0%	15.1%	56.7%	0%
Taking elevator		0.9%	84.4%	0.2%	1.8%	12.8%	0%
Climbing stairs		9.2%	1.8%	1.4%	5.3%	82.4%	0%
Idle		0%	0%	0%	19.1%	20.9%	0%
Driving		2.9%	0.7%	0.1%	14.7%	81.6%	0%
Door closing		1.1%	0.5%	0%	0%	2.7%	95.7%
